# Intracranial Hypotension Following Spinal Manipulation: A Case Report and Scoping Review of the Literature

**DOI:** 10.1002/brb3.71409

**Published:** 2026-04-27

**Authors:** Marina Romozzi, Matteo Palermo, Fabio Zeoli, Federico Tosto, Catello Vollono, Giuseppe Garignano, Francesco Signorelli

**Affiliations:** ^1^ Department of Neuroscience Università Cattolica del Sacro Cuore Rome Italy; ^2^ Neurology Unit, Dipartimento di Neuroscienze, Organi di Senso e Torace Fondazione Policlinico Universitario Agostino Gemelli IRCCS Rome Italy; ^3^ Department of Neurosurgery, Fondazione Policlinico Universitario Agostino Gemelli IRCCS Università Cattolica del Sacro Cuore Rome Italy; ^4^ Department of Neuroscience “Giovanni Paolo II” Hospital Lamezia Terme Italy; ^5^ Radiology and Neuroradiology Unit, Dipartimento di Diagnostica per Immagini, Radioterapia Oncologica e Ematologia. Fondazione Policlinico Universitario Agostino Gemelli IRCCS Rome Italy

**Keywords:** Chiropractic, Intracranial hypotension, Leak, Osteopathy

## Abstract

**Purpose:**

Spinal manipulative therapies, including chiropractic and osteopathic maneuvers, are widely practiced for musculoskeletal complaints. However, rare but serious complications such as cerebrospinal fluid (CSF) leak with subsequent intracranial hypotension (IH) have been described. The pathophysiological mechanism is presumed to involve mechanical stress on the spinal dura during high‐velocity movements, leading to dural tears, particularly in the cervicothoracic region.

**Methods:**

We conducted a scoping review in accordance with the PRISMA extension for Scoping Reviews (PRISMA‐ScR) guidelines, through a comprehensive search of PubMed and Scopus. We complemented the review with an illustrative case from our institution.

**Results:**

We identified 21 eligible studies, including 21 patients with IH following spinal manipulation. Most patients were women (81%), aged 29–54 years, and the majority underwent cervical maneuvers. Symptom onset was typically within the first week, and all presented with orthostatic headache, often accompanied by nausea, neck pain, tinnitus, or visual disturbances. Neuroimaging consistently revealed features of IH, with pachymeningeal enhancement and subdural collections as the most frequent findings; spinal imaging frequently demonstrated extradural CSF collections. Management was conservative in about one‐third of cases, but most required epidural blood patching, which was effective in the majority. Surgical repair was necessary in rare, refractory cases, particularly in the presence of structural spinal abnormalities. Overall prognosis was favorable, with 95% of patients achieving full recovery. Our illustrative case highlights the potential for severe complications such as subdural hematomas and recurrence if the underlying leak is not addressed.

**Conclusion:**

Clinicians should recognize the possibility of CSF leaks after spinal manipulation, especially in patients with new‐onset orthostatic headache.

## Graphical abstract

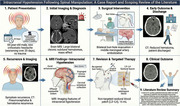

## 1 Introduction

Spinal manipulative therapies, including chiropractic and osteopathic high‐velocity low‐amplitude adjustments, are widely practiced for musculoskeletal complaints (Vickers and Zollman 1999). While generally considered safe, serious complications can occur. One such infrequent but notable complication is the development of a cerebrospinal fluid (CSF) leak leading to intracranial hypotension (IH) (Kusnezov et al. 2013).

The presumed pathophysiology underlying post‐manipulation CSF leaks is a tear or defect in the spinal dura mater induced by mechanical forces. High‐velocity neck or back manipulations involve rapid rotational or flexion–extension movements that can subject the spinal elements to abrupt stress. It is postulated that an overly forceful or improperly directed thrust could produce a small dural tear, especially at vulnerable sites (Kusnezov et al. 2013). Indeed, forceful cervical flexion/extension is an accepted mechanism for traumatic dural rupture leading to CSF leakage. Such leaks are often ventral in location and in the cervicothoracic region, which aligns with the typical distribution of spontaneous CSF leaks as well (Kusnezov et al. 2013). Underlying structural factors may increase susceptibility: for example, degenerative spine changes like osteophytes or calcified disc extrusions can erode or weaken the dura (Kusnezov et al. 2013).

Spontaneous intracranial hypotension (SIH) caused by CSF volume depletion classically presents with orthostatic headache that worsens upon standing and improves when supine. In the context of spinal manipulation, a growing body of case reports since the early 2000s has documented patients developing SIH shortly after cervical or thoracic spine adjustments (Kusnezov et al. 2013; Ernst 2007). These reports, though rare, have raised awareness that forceful spinal mobilization may precipitate a CSF leak in susceptible individuals (Yin et al. 2014).

Herein, we report a case of CSF leak following chiropractic spinal manipulation and provide a scoping review of the proposed mechanisms, clinical presentation, imaging features, illustrative cases, and management strategies for CSF leaks and IH associated with spinal manipulation.

## 2 Methods

We conducted a scoping review according to the PRISMA‐ScR (preferred reporting items for systematic reviews and meta‐analyses extension for scoping reviews) guidelines published in 2018 (Tricco et al. 2018). A protocol was not prospectively registered due to the descriptive, case‐based nature of the evidence.

We also present an illustrative case of post‐manipulative IH from our cohort of patients at Fondazione Policlinico Universitario A. Gemelli IRCCS in Rome.

### 2.1 Search Strategy

Three authors (M.R., M.P., and F.Z.) performed a comprehensive search of the PubMed/MEDLINE and Scopus databases to identify studies reporting cases of CSF leak or IH occurring after spinal manipulation maneuvers. The following search strategy was applied: (*“Intracranial Hypotension” OR “Spontaneous Intracranial Hypotension”*) *AND* (“Chiropractic Manipulation” OR “Spinal Manipulation” OR “Osteopathic Manipulation” OR Chiropractic OR Osteopathic OR Osteopathy OR Osteopath*). The search was limited to articles published in English and was updated to August 9, 2025. A forward citation search of the references from all retrieved articles was also conducted to increase the search power.

### 2.2 Study Selection

We included studies that reported cases of CSF leak and/or IH occurring after spinal manipulation maneuvers, including chiropractic manipulation, osteopathic manipulation, or other manual spinal mobilization techniques. For the purposes of this study, only cases with a clear temporal relationship between the manipulation procedure and the onset of symptoms, and with diagnostic confirmation through neuroimaging and/or lumbar puncture (LP), were included. We excluded cases in which the CSF leak was attributable to other identifiable causes like neurosurgical procedures, penetrating trauma, spinal anesthesia, or spontaneous leaks without preceding manipulation. Animal and preclinical studies were excluded, as were review articles, conference abstracts, and studies lacking sufficient clinical or diagnostic details.

Two authors (M.R. and M.P.) independently screened the titles and abstracts of all articles identified through the search strategy and selected studies according to the previously listed inclusion and exclusion criteria. Discrepancies were resolved through discussion until a consensus was reached.

**FIGURE 1:**
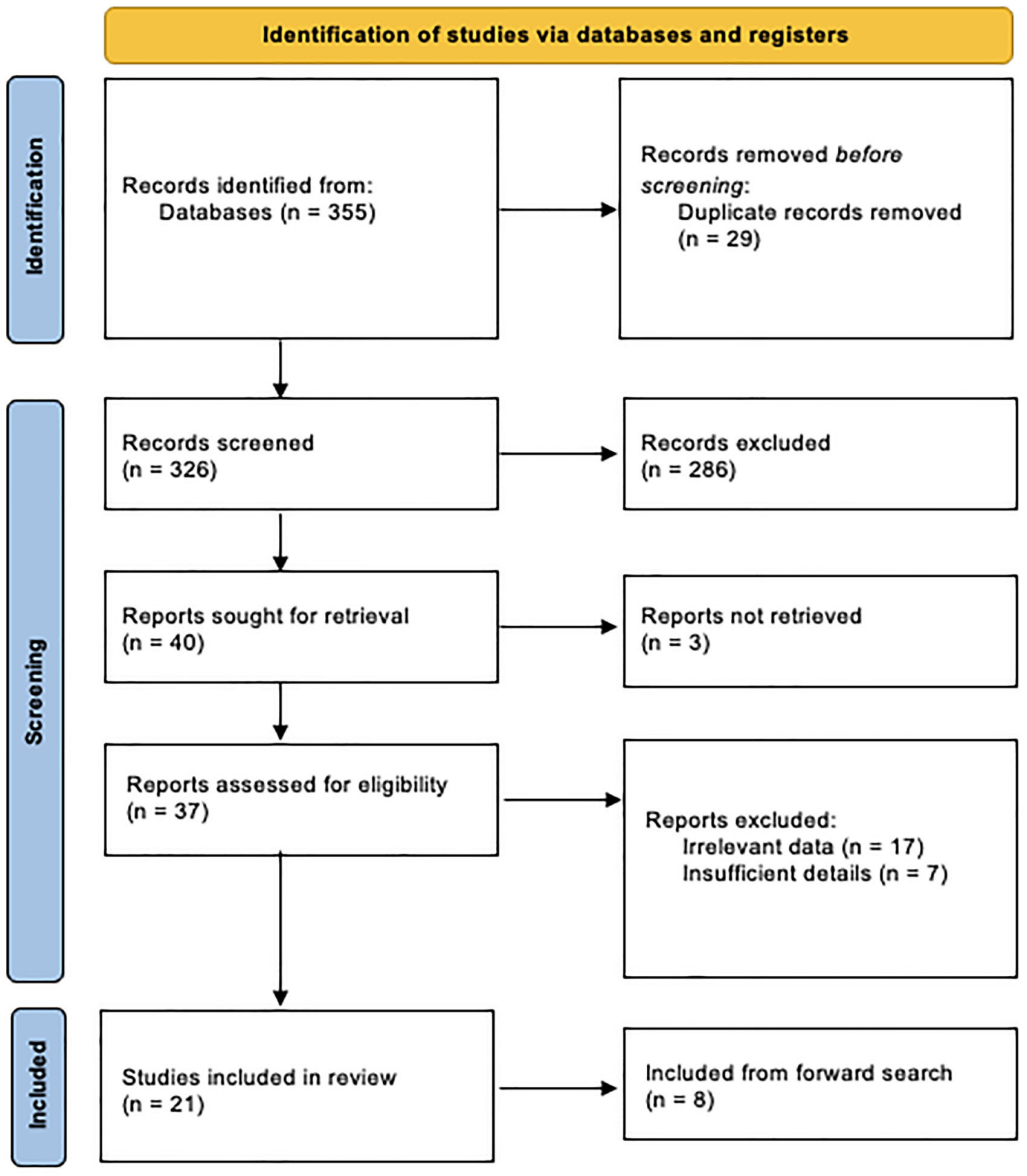
PRISMA extension for scoping reviews (PRISMA‐ScR) flowchart of study selection process.

After excluding ineligible articles, the full texts of the remaining studies were reviewed to confirm eligibility using the same criteria (Figure 1). Any disagreements were resolved during a consensus meeting through joint reassessment of the article and the extracted data (F.S., M.R., M.P., F.Z.).

### 2.3 Data Charting

Data were charted using a standardized form developed a priori and piloted on a subset of included reports. For each eligible study, we extracted the author and year of publication, number of patients, spinal manipulative therapy (SMT) technique used, and the interval between SMT and symptom onset. Additionally, we gathered information on the age of the patients at diagnosis, sex, clinical presentation, and opening pressure. Regarding diagnosis, we collected data on the type of cranial and spinal imaging performed and the treatment strategy implemented (Table 1). We also sought to document the method used for CSF leak detection, as well as information on both unsuccessful and successful treatment attempts.

**TABLE 1:** Cases with intracranial hypotension following chiropractic manipulation.

Authoryear	Age/sex	SMT technique	Interval after SMT	Clinical presentation	Opening pressure (LP)	Cranial imaging findings	Spinal MRI findings	CSF leak detection procedure	Management/Outcome
Chung et al. 2000	44/F	Not described	Not described	Orthostatic headache, neck stiffness	Not mentioned	Not mentioned	Not mentioned	RC: no CSF leak	Supportive/complete
Jeret 2001	34/M	Rapid rotatory neck manipulation	36 h	Orthostatic headache, neck pain, dizziness	80 mm H_2_O	Plain CT: normal, MRI + C: normal	None	Myelography (after 5 weeks): no CSF leak	EBP x1/Partial
Beck et al. 2003	40/F	Axial tension and rotation	Within 24 h	Orthostatic headache, nausea and vomiting, doubling of vision	Not mentioned	MRI + C: diffuse PE, MRI: bilateral subdural effusion	Ax T2 Cervical: CSF accumulation in dorsal perivertebral space around the dural sac at C1–C2 level	None	Supportive/complete
Suh et al. 2005	37/F	Axial tension and rotation	1 day	Orthostatic headache, nausea and vomiting, neck pain	40 mm H_2_O	CT + C: diffuse PE, MRI + C: PE	Sag T2 Thoracic: epidural and subdural fluid collection, Sag + Thoracic and Lumbar + C: diffuse dural enhancement and epidural venous engorgement	None	Initially supportive then EBP x1, 20 mL/complete
Strauss et al. 2005	54/F	Axial tension and rotation	Immediate	Orthostatic headache, vomiting	30 mm H_2_O	MRI: bilateral PE	None	RC: CSF leak upper thoracic level	Initially supportive, then EBP x1, 20 mL/complete
Mathews et al. 2006	51/F	Not described	7 days	Headache, diplopia	No LP	MRI: bilateral PE	None	Myelography: CSF leak at C2 (Strauss et al. 2005)	EBP x1/complete
Morelli et al. 2006	49/M	Rotation and hyperextension	1 day	Orthostatic headache, tinnitus	No LP	Plain CT: normal, MRI + C: fronto‐parietal PE	MR whole spine + myelography	MR myelography: arachnoid cyst of third cervical root + CSF leakage	Supportive/complete
Prasad et al. 2006	37/F	Axial tension and rotation	Immediate	Orthostatic headache	Not mentioned	Plain CT: bilateral subdural hygroma; tonsillar herniation	Cervical: extra‐axial fluid collection	Planar scintigraphy: abnormal radiotracer activity at anterior thoracolumbar spine	EBP x1/Complete
Donovan et al. 2007	32/F	Cervical and thoracic mobilization	Immediate	Orthostatic headache, nausea, photophobia, left C8 radicular pain with weakness left‐sided hearing loss	< 5 mm H_2_O	Brain MRI: diffuse PE	MRI: epidural and subdural CSF collections, epidural venous engorgement, diffuse dural enhancement	RC: no visible leak. CT myelogram: large leak from C8 to T5.d	Initially conservative treatment, then targeted EBP x2/complete
Kurbanyan and Lessell 2008	46/F	Axial tension and rotation	Not specified	Orthostatic headache, neck stiffness, diplopia	Not mentioned	None	Sag cervical + C: enhancement of basilar meninges	None	Supportive/complete
Wagner et al. 2010	42/F	Thoracic spinal manipulation	2 days	Orthostatic occipital headache, tinnitus, slight neck stiffness	40 mm H_2_O	MRI: no typical signs of intracranial hypotension; subdural hemorrhage excluded	T2 fat‐sat: left root‐sheath cysts at Th10/11 and Th12/L1	MR myelography: leaks at left T3/4 and T10/11	EPB x1/complete
Chen et al. 2011	33/M	Cervical manipulation	Immediate	Orthostatic headache	Not reported	CT: bilateral chronic SDH. MRI brain: diffuse PE, obliteration of basal cisterns, SDH	MRI: CSF accumulation/leak around C1–C2 with paraspinal fluid	None	Supportive/complete
Kusnezov et al. 2013	29/F	Axial tension and rotation	1 week	Orthostatic headache, nausea and vomiting	None	None	Ax T2 Cervical: ventral C6 epidural fluid	None	Supportive/complete
Ahn et al. 2013	49/F	Cervical manipulation	2 days	Orthostatic headache, nausea/vomiting, bilateral tinnitus	80–90 mm H_2_O	MRI: diffuse PE+ brain sagging	Not reported	RC: leak at T1; additional at C3 and T6–T7	EBP x1/complete
Wilson et al. 2015	32/F	Axial tension and lateral flexion	Not specified	Orthostatic headache, dizziness, vomiting, blurring of vision	None	MRI: flattening of pons, cerebellar tonsil descent and PE	MRI: C5/6 central disc protrusion indenting the thecal sac and a ventral epidural CSF collection extending from C6 to T7	None	C5/6 anterior cervical discectomy; removal of calcified disk fragment; PEEK implant/complete
Hawson and Taylor 2015	36/F	Not reported	1 day	Orthostatic headache, pulsatile tinnitus, nausea, vomiting, neck and back pain, bilateral hand paraesthesias	Not reported	MRI brain: no subdural fluid, no venous sinus engorgement, no brain sagging	MRI spine: no apparent CSF leak	None	Initially conservative treatment, then EBP x1/complete
Lin and Weng 2017	36/F	Tension on occipital area, posterior nuchal area, and bilateral shoulders	1 day	Orthostatic headache	5 mm H_2_O	MRI + C: brain sagging, cerebellar hemispheres PE + bilateral engorgement of transverse sinuses	Spine MRI: periradicular CSF leaks, dural tear at CT‐T1 junction, marked CSF leakage near bilateral apical pleural regions	MRI myelography: symmetric SPFs permitted flow of CSF into pleural cavities over bilateral lungs	Targeted EBP x3/complete
Fernando et al. 2022	36/F	Axial tension and rotation	2 weeks	Orthostatic headache, dizziness, nausea and double vision	None	CT + C: subdural hemorrhage, interhemispheric MRI + C: diffuse PE; sagging brain signs	Sag STIR Cervical: hyperintense signal in interspinous region at C1–C2 level	None	Supportive/complete
Sutton et al. 2022	34/M	Cervical manipulation	1 day	Orthostatic headache, nausea	Not reported	Low‐lying cerebellar tonsils; prominent dural venous sinuses	Epidural CSF isodense fluid from C4 to C7 around the dural sac	CT myelogram	EBP performed/complete.
Bozer et al. 2024	40/F	Thoracic spinal manipulation	4 days	Orthostatic headache, nausea, vomiting, intractable symptoms	9 cm H_2_O	Diffuse ME, pituitary enlargement, venous sinus distension, small subdural hematomas	Extradural CSF collection through canal, pooling at T6; later dynamic imaging suggested leak at T11–T12	CT myelogram	EBP at T6–T7/Partial
									EBP at T11–T12/Partia
									lT11–T12 laminectomy and dural repair/complete
Kim 2024	30/F	Manual cervical adjustment with axial tension + neck rotation/extension	5 days	Orthostatic headache	55 cm H_2_O	Bilateral subdural effusions, diffuse PE; slit‐like ventricles	Not reported	RC: extradural tracer accumulation at upper cervical level	Initially conservative therapy, then targeted EBP x1/complete

Abbreviations: Ax T2, axial T2‐weighted MRI; CSF, cerebrospinal fluid; CT + C, computed tomography with contrast; EBP, epidural blood patch; LP, lumbar puncture; ME, meningeal enhancement; MRI + C, magnetic resonance imaging with contrast; PE, pachymeningeal enhancement; RC, radioisotope cisternography; Sag T2, sagittal T2‐weighted MRI; SMT, spinal manipulative therapy; SPFs, spinal pleural fistulas; STIR, short tau inversion recovery.

### 2.4 Risk of Bias

Consistent with scoping review methodology, we did not perform a formal risk‐of‐bias assessment; instead, we mapped available evidence and highlighted limitations inherent to case reports.

#### 2.4.1 Case Description

A 65‐year‐old patient without a previous history of headache presented with a progressively worsening headache, with orthostatic features, poorly responsive to medical therapy, that has lasted for the past 20 days. The patient denied any recent trauma.

He reported having undergone cervical osteopathic manipulations within the past 3 months for recurrent cervicalgia. A brain MRI without contrast was performed, showing a large bilateral subdural hematoma with significant mass effect on the cortical gyri. The patient was admitted to the emergency department and underwent neurosurgical evacuation of a bilateral chronic subdural hematoma via burr holes. Subsequently, endovascular embolization of the middle meningeal arteries was performed as an adjunctive treatment to reduce the risk of recurrence.

The surgical procedure was performed without complications. A cranial CT scan showed a reduction in the volume of the hematoma. Therefore, the patient was discharged. However, after a transient improvement in the symptoms, the patient continued to present a fluctuating headache without positional features, with four to five episodes per month. He was readmitted to our clinic and, upon arrival at the ER, a head CT scan showed an increase in pneumocephalus and a recurrence of the hematoma.

**FIGURE 2:**
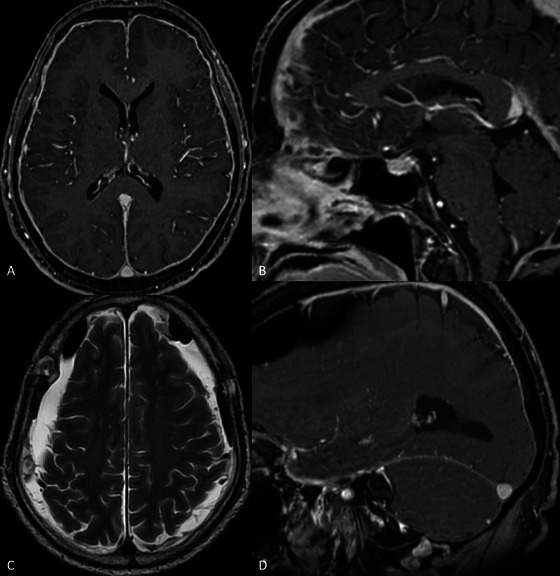
Brain magnetic resonance imaging (MRI) showing signs of intracranial hypotension. (Panel A) Axial post‐contrast 3D FSPGR image showing diffuse pachymeningeal enhancement, a hallmark sign of intracranial hypotension due to compensatory meningeal vascular engorgement. (Panel B) Sagittal post‐contrast 3D FSPGR image demonstrating signs of brain sagging, with reduction of the mammillopontine distance, as well as effacement of the prepontine and suprasellar cisterns. (Panel C) Axial T2‐weighted image showing bilateral subdural HYPERINTENSE fluid collections with air‐fluid levels following surgical evacuation via fronto‐parietal burr holes. (Panel D) Sagittal post‐contrast 3D FSPGR image depicting engorgement of the right transverse sinus, reflecting venous distension secondary to low intracranial pressure.

The following day, an MRI of the neuraxis with contrast was performed, which revealed radiological findings suggestive of IH: pachimeningeal enhancement, subdural fluid collection, dural venous engorgement, cervical spinal longitudinal extradural collection, and effacement of the suprasellar cistern. The Bern score was 7 (Figure 2; Supplementary Figure 1).

Given these findings, a surgical revision of the previous burr holes was performed without periprocedural complications.

After the first day, a non‐targeted epidural blood patch (EBP) was performed under local anesthesia by injecting 16 mL of autologous blood into the L3–L4 epidural space, using a technique already described by our group (Romozzi et al. 2025). The procedure was uneventful. A cranial CT scan showed satisfactory surgical outcomes, highlighting a reduction in the volume of the hematoma and of the pneumoencephalus. The patient was subsequently discharged with complete resolution of the headache.

## 3 Results

We performed a descriptive synthesis. Findings were summarized using counts and proportions where appropriate and organized into specific thematic domains (clinical presentation, imaging patterns, leak localization, management strategies, complications, and outcomes). Given the heterogeneity of the evidence based on case reports and case series, no meta‐analysis was planned. The initial search retrieved 355 records from PubMed and Scopus. Before screening, 28 non‐English records were excluded. During the screening phase, 286 records were excluded for not meeting the inclusion criteria, most commonly because they reported unrelated conditions (e.g., spontaneous CSF leaks without preceding manipulation, vascular injuries, or musculoskeletal outcomes only), were review articles, conference abstracts, or animal/preclinical studies. Of the 41 reports assessed for eligibility, 17 were excluded for irrelevant or insufficient data, three reported patients with a leak attributable to another cause, and seven were excluded for insufficient details. Seven studies were included from the forward search. Ultimately, 21 studies were included in the final analysis (Figure 1; Table 1). The study selection process was documented according to the PRISMA‐ScR, with a flow diagram illustrating the phases of identification, screening, eligibility assessment, and final inclusion (Figure 1).

### 3.1 Qualitative Analysis

A total of 21 studies published between 2002 and 2025 were included, including 21 patients diagnosed with IH following spinal manipulation maneuvers (Table 1). Among 21 reported cases, 17 were females (81%), and four were males (19%), ranging from 29 to 54 years old (Kusnezov et al. 2013; Chung et al. 2000; Jeret 2001; Beck et al. 2003; Suh et al. 2005; Strauss et al. 2005; Mathews et al. 2006; Morelli et al. 2006; Prasad et al. 2006; Donovan et al. 2007; Kurbanyan and Lessell 2008; Wagner et al. 2010; Ahn et al. 2013; Wilson et al. 2015; Hawson and Taylor 2015; Lin and Weng 2017; Fernando et al. 2022; Sutton et al. 2022; Bozer et al. 2024; Kim 2024). Because the available evidence consists predominantly of case reports/series without incidence estimates, it was not feasible to provide incidence estimates; therefore, we report the number and characteristics of published cases and highlight evidence gaps.

SMT techniques vary, most often involving high‐velocity cervical maneuvers. The most frequent were axial tension with rotation in seven cases (33.3%), unspecified cervical manipulation in four cases (19%), and thoracic spinal manipulation in two cases (9.5%). Less common single‐case techniques included rotation with hyperextension, combined cervical and thoracic mobilization, axial tension with lateral flexion, and occipital/shoulder tension technique (*n* = 1 case each). Three cases (13%) did not report the exact method used (Chung et al. 2000, Kurbanyan and Lessell 2008, Wilson et al. 2015).

Symptom onset after SMT ranged from immediately to 2 weeks. Immediate onset occurred in four cases (19%), within 1 day in six cases (28.6%), and within the first week in seven cases (33.3%), including one case at 36 h, two cases at 2 days, two at 4–5 days, and two at 1 week. Onset > 1 week was reported in one case (4.5%), while the interval was not specified in three cases (13.7%) (Kusnezov et al. 2013, Chung et al. 2000, Jeret 2001, Beck et al. 2003, Suh et al. 2005, Strauss et al. 2005, Mathews et al. 2006, Morelli et al. 2006, Prasad et al. 2006, Donovan et al. 2007, Kurbanyan and Lessell 2008, Wagner et al. 2010, Ahn et al. 2013, Wilson et al. 2015, Hawson and Taylor 2015, Lin and Weng 2017, Fernando et al. 2022, Sutton et al. 2022, Bozer et al. 2024, Kim 2024, Chen et al. 2011).

All patients explicitly noted headache, and orthostatic or postural headache was reported in 20 cases (Kusnezov et al. 2013, Chung et al. 2000, Jeret 2001, Beck et al. 2003, Suh et al. 2005, Strauss et al. 2005, Mathews et al. 2006, Morelli et al. 2006, Prasad et al. 2006, Donovan et al. 2007, Kurbanyan and Lessell 2008, Wagner et al. 2010, Ahn et al. 2013, Wilson et al. 2015, Hawson and Taylor 2015, Lin and Weng 2017, Fernando et al. 2022, Sutton et al. 2022, Bozer et al. 2024, Kim 2024, Chen et al. 2011). Other common symptoms included: nausea and/or vomiting in 11 cases (52.4%), neck pain/stiffness in six cases (28.6%), tinnitus in four cases (19%), dizziness in three cases (14.3%), and visual disturbances in five patients (23.8%). Less frequent features were photophobia in one case, transient hearing loss in one case, and radicular symptoms in one case.

LP opening pressure was reported in nine of 21 cases (42.9%) (Jeret 2001, Suh et al. 2005, Strauss et al. 2005, Donovan et al. 2007, Wagner et al. 2010, Wilson et al. 2015, Lin and Weng 2017, Bozer et al. 2024, Kim 2024). Values were generally low, ranging from ≤ 5 mm H_2_O in one case to 40 mm H_2_O, consistent with IH. Two cases had borderline low‐normal pressures (80–90 mm H_2_O), and one outlier was documented at 55 mmH_2_O, which deviated from the typical low‐pressure pattern.

Most patients had characteristic intracranial MRI/CT findings of IH.

The most frequent imaging finding was diffuse pachymeningeal enhancement, observed in 12 cases (57.1%) (Beck et al. 2003, Suh et al. 2005, Strauss et al. 2005, Mathews et al. 2006, Wagner et al. 2010, Ahn et al. 2013, Lin and Weng 2017, Fernando et al. 2022, Bozer et al. 2024, Kim 2024, Chen et al. 2011). MRI typically demonstrated dural thickening with marked contrast enhancement in the majority of patients.

Less frequent findings included subdural fluid collections or effusions (five cases, 23.8%), brain “sagging” or tonsillar herniation (six cases, 28.6%), or engorgement of venous sinuses and pituitary gland enlargement (one case). Importantly, two early onset cases (9.5%) had normal initial cranial imaging despite classic clinical features of IH (Jeret 2001, Hawson and Taylor 2015). However, the vast majority showed one or more of the above abnormalities on post‐contrast MRI.

Spinal MRI was performed in most reported cases, with abnormalities consistent with CSF leakage identified in 14 of 21 patients (66.7%) (Kusnezov et al. 2013, Chung et al. 2000, Beck et al. 2003, Suh et al. 2005, Morelli et al. 2006, Prasad et al. 2006, Donovan et al. 2007, Kurbanyan and Lessell 2008, Wagner et al. 2010, Wilson et al. 2015, Hawson and Taylor 2015, Lin and Weng 2017, Fernando et al. 2022, Sutton et al. 2022, Bozer et al. 2024, Chen et al. 2011). The most common finding was epidural CSF collection, present in approximately nine patients (42.5%), characterized by extradural fluid tracking along the spinal cord. Additional common features included dural thickening/enhancement and epidural venous engorgement, frequently involving the thoracic spine. Meningeal diverticula or cysts were noted in a minority of cases, including small nerve root sleeve cysts on fat‐saturated T2 sequences and, in one patient, bilateral pleural CSF fistulae at the lung apices. Although spinal MRI often demonstrated indirect signs suggestive of the leak site, it rarely provided precise localization. Overall, in eight cases (38.1%), spinal MRI was normal or nondiagnostic, requiring further targeted imaging for leak detection (Jeret 2001, Strauss et al. 2005, Mathews et al. 2006, Morelli et al. 2006, Kurbanyan and Lessell 2008, Hawson and Taylor 2015, Fernando et al. 2022, Bozer et al. 2024).

To localize the exact site of CSF leakage, several diagnostic modalities were utilized (Chung et al. 2000, Jeret 2001, Strauss et al. 2005, Mathews et al. 2006, Morelli et al. 2006, Prasad et al. 2006, Donovan et al. 2007, Wagner et al. 2010, Ahn et al. 2013, Lin and Weng 2017, Sutton et al. 2022, Bozer et al. 2024, Kim 2024).

CT myelography was performed in four patients (19%) and successfully identified the leak in all cases. MR myelography, using heavy T2‐weighted or gadolinium‐enhanced techniques, was reported in three patients (14.3%), all yielding positive results, including identification of leaks at T3–T4 and T10–T11, as well as demonstration of rare bilateral spinal‐pleural CSF fistulas. Radionuclide cisternography was used in five patients (23.8%) with variable diagnostic yield: half localized a leak, while the remainder were negative or indeterminate, prompting further imaging. A variant, planar scintigraphy, in one case demonstrated abnormal tracer uptake in the thoracolumbar region (Prasad et al. 2006). Conventional myelography was performed in a single patient 5 weeks after symptom onset, but failed to reveal a leak, and was otherwise largely replaced by more advanced modalities in these reports. In total, leak sites were identified in roughly 70% of all cases, either via MRI or the specialized studies, most often in the cervical or upper thoracic spine (Jeret 2001, Strauss et al. 2005, Mathews et al. 2006, Morelli et al. 2006, Prasad et al. 2006, Donovan et al. 2007, Wagner et al. 2010, Ahn et al. 2013, Lin and Weng 2017, Sutton et al. 2022, Bozer et al. 2024, Kim 2024).

Management ranged from conservative care to interventions, with excellent overall outcomes. Standalone conservative management was sufficient only in seven patients (33.3%); thus, all seven recovered completely (Kusnezov et al. 2013, Chung et al. 2000, Beck et al. 2003, Morelli et al. 2006, Kurbanyan and Lessell 2008, Fernando et al. 2022, Chen et al. 2011). These were typically cases with milder symptoms or where imaging did not show a definite treatable leak, and spontaneous resolution occurred with conservative care.

Note that 13 patients (61.9%) underwent one or more EBPs to seal the dural defect (Chung et al. 2000, Jeret 2001, Suh et al. 2005, Strauss et al. 2005, Mathews et al. 2006, Prasad et al. 2006, Donovan et al. 2007, Wagner et al. 2010, Ahn et al. 2013, Wilson et al. 2015, Hawson and Taylor 2015, Lin and Weng 2017, Bozer et al. 2024). Most (*n* = 10 cases) required a single EBP, resulting in complete symptom resolution. In three cases, multiple EBPs were needed. Of the EBP‐treated patients, 13/14 achieved complete or near‐complete recovery. Only one case had lingering symptoms described as partial improvement after one EBP. Notably, even when initial EBP provided only partial relief, repeat or higher volume patches often led to full recovery in subsequent attempts.

Surgical repair was rarely necessary: in fact, it was performed only in two cases (9.5%) (Wilson et al. 2015, Bozer et al. 2024). One patient with a cervical disc‐related dural tear underwent surgical disc removal and dural repair, which cured the hypotension headache. Another patient required a thoracic laminectomy with direct dural repair after two failed blood patches, after which she fully recovered. All patients who underwent surgery had complete resolution of symptoms.

Overall, 20 of 21 patients (95.2%) achieved complete recovery by final follow‐up. Only one case had a less‐than‐complete outcome. There were no deaths or permanent neurological deficits reported (Jeret 2001).

## 4 Discussion

This scoping review maps the published case‐based evidence of potential association between SMT and the development of IH. However, the precise incidence of this complication is unknown, and the available literature consists predominantly of isolated case reports and small case series, which limits causal inference and precludes any estimation of incidence (Fernando et al. 2022).

A review of published cases up to 2014 identified eight case reports worldwide linking spinal manipulation to IH (Tuchin 2014). These data altogether may suggest that this complication is extremely uncommon. However, some authors have pointed out that the true incidence may be slightly higher than reported, since milder leaks could go unrecognized as the patient's headache might resolve with rest and never come to medical attention (Prasad et al. 2006, Donovan et al. 2007, Wagner et al. 2010, Tuchin 2014).

One of the earliest documented cases appeared in 2003: a patient developed intractable orthostatic headaches after a cervical chiropractic manipulation, and imaging confirmed IH due to a spinal CSF leak (Chung et al. 2000).

Over the subsequent years, a handful of similar cases emerged in the literature. Notable reports have come from neurology, neurosurgery, and radiology journals. For instance, Suh et al. (2005) reported a 36‐year‐old woman who experienced a throbbing orthostatic headache with nausea immediately after a cervical spine manipulation; her brain MRI showed diffuse pachymeningeal enhancement, and spinal MRI revealed a cervicothoracic CSF leak, which was successfully treated with an EBP. Around the same time, Strauss et al. (2005) described a post‐manipulation IH case in a middle‐aged woman. Morelli et al. (2006) documented a case with MRI‐visible dural leakage at the cervical level after chiropractic treatment, emphasizing that even though arterial dissections are more commonly discussed, practitioners should also be aware of IH as a potential complication (Mathews et al. 2006, Romozzi et al. 2025).

In 2008, Kurbanyan and Lessell (2008) reported a patient who developed an isolated sixth cranial nerve palsy after upper cervical manipulation, ultimately attributed to IH; the neurological deficit resolved spontaneously over time, paralleling closure of the CSF leak. This case underscored that IH can have atypical presentations, including focal cranial nerve deficits without prominent headache, and still be temporally linked to recent spinal manipulation. In 2013, Kusnezov et al. (2013) published a case unique for providing serial MRI evidence of the condition's evolution: a 29‐year‐old woman developed orthostatic headache 1 week after a neck “axial tension and rotatory manipulation”. The initial MRI demonstrated a new ventral CSF collection at the C5–C6 level, while follow‐up imaging several months later confirmed a reduction of the leak after conservative management. This provided one of the first clear imaging documents of the potential link between this manipulation and CSF leak (Kusnezov et al. 2013).

In subsequent years, a few additional reports have further suggested this plausible association. Wilson et al. (2015) presented the case of a calcified cervical disc herniation that likely lacerated the dura during a chiropractic adjustment, producing persistent CSF leakage that did not respond to patches and ultimately required surgical repair of the dura (Wilson et al. 2015). This case illustrates how an occult structural lesion combined with manipulation led to a complex scenario of SIH. More recently, Bozer et al. reported a case following a thoracic spine manipulation, which was different from all prior cases that involved the cervical region. In their case, the initial MRI was unable to definitively locate the leak, but advanced imaging identified a thoracic dural defect. The patient underwent multiple EBPs, including targeted, image‐guided patches with fibrin sealant, without lasting success, and finally, surgical exploration was needed to seal the leak (Bozer et al. 2024). This represents the most intensive level of intervention reported for a post‐manipulation CSF leak, reinforcing that, although rare, the complication can pose significant management challenges.

It is worth noting that not all reported cases explicitly involved chiropractors; some occurred after SMTs performed by other practitioners like osteopathic physicians or manual physical therapists. A 2022 systematic review by Fernando et al. found that in about five of 14 cases identified, the provider was not a chiropractor (Ernst 2007, Tuchin 2014, Chiapparini et al. 2004). This suggests the phenomenon is tied to the manipulation maneuver itself rather than the specific discipline.

In summary, the literature to date consists of scattered case reports describing IH temporally associated with a recent spinal mobilization. While causality in each instance cannot be absolutely proven, the consistency of clinical presentation and the plausible mechanism lend credence to a cause‐and‐effect relationship. The rarity, however, means that these are idiosyncratic events possibly precipitated by unique patient vulnerabilities in conjunction with spinal manipulation.

### 4.1 Clinical Presentation and Diagnostic Criteria

Patients who develop a CSF leak after spinal manipulation typically present with symptoms indistinguishable from SIH (Yin et al. 2014, Fernando et al. 2022, Chiapparini et al. 2004). The hallmark is a severe postural headache, often described as a diffuse, pulling, or pressure‐like pain that is triggered or worsened by upright posture and relieved on lying flat. Common accompanying features include neck stiffness or pain, nausea, vomiting, vertigo, and auditory changes, such as muffled hearing or tinnitus. Visual disturbances can also occur. Notably, cranial nerve palsies are a known but less common manifestation of IH (Yin et al. 2014, Fernando et al. 2022, Chiapparini et al. 2004). These symptoms usually begin within hours to days after the manipulation. In some reported cases, the onset was almost immediate, whereas in others the headache developed after a delay of several days up to a week, presumably as a slow CSF leak gradually led to intracranial volume depletion (Beck et al. 2003, Mathews et al. 2006, Kurbanyan and Lessell 2008, Wilson et al. 2015, Fernando et al. 2022). Given this timing, patients might not always link the headache to a spine treatment unless specifically asked with a thorough clinical history.

Diagnostically, postural headache in this setting should prompt evaluation for IH. Gadolinium‐enhanced brain MRI is the noninvasive imaging modality of choice to support the diagnosis. Characteristic MRI findings include the following: diffuse pachymeningeal enhancement and signs of brain sagging (Chiapparini et al. 2004). Sagging refers to downward displacement of brain structures due to loss of buoyant support, and it may be evidenced by tonsillar herniation, enlargement of venous sinuses with engorgement, subdural fluid collections, and pituitary gland enlargement from venous engorgement (Ahn et al. 2013, Hawson and Taylor 2015, Lin and Weng 2017). These intracranial changes reflect low CSF pressure and volume. In one illustrative case, after a chiropractic manipulation, the patient's brain MRI showed diffuse pachymeningeal enhancement, consistent with IH, and her LP yielded essentially a “dry tap” (Suh et al. 2005).

The report by Beck et al. (2003) that a spinal chiropractic manipulation may cause IH opens the debate between external/epigenetic and internal/genetic factors in the etiology of dural tears. In fact, those risk factors should be taken into account; from the external perspective, manipulation could produce mechanical stress on the dural sac; however, manual neck movements generate only a fraction of the force required to fracture bone, making it difficult to explain how they could rupture a “healthy” dura. This raises the possibility of an intrinsic predisposition due to hereditary connective tissue disorders (Bascom et al. 2019).

In this context, Beck et al. (2003) highlighted a frequent presence of marfanoid features in young chiropractic patients, including arm span/height ratio > 1.03; pectus excavatum; scoliosis; skin striae; and long, thin fingers. Microfibrillinopathy appears relatively common among patients with CSF leakage, and only about 20% of basilar skull fractures with a dural tear actually result in a CSF leak, further supporting the role of intrinsic dural vulnerability (Beck et al. 2003). However, none of the patients in our analysis were reported as carrying a hereditary connective tissue disorder of any type.

### 4.2 Localization of the Leak

Once IH is recognized, localizing the CSF leak is the next diagnostic challenge. Routine spinal MRI can sometimes identify telltale signs of the leak, such as extra‐arachnoid fluid collections tracking along the spinal epidural space (Morelli et al. 2006, Donovan et al. 2007, Wagner et al. 2010, Tuchin 2014). In post‐manipulation cases reported, MRI of the spine often revealed an extradural CSF collection at the level of injury. For example, one patient's cervical MRI showed a ventral epidural fluid collection extending from C5–C6 into the upper thoracic levels without significant cord compression (Kusnezov et al. 2013). However, standard MRI may not definitively pinpoint small dural defects. If clinical suspicion remains high and initial imaging is non‐localizing, specialized studies are indicated. Heavily T2‐weighted MR myelography, CT myelography, or digital subtraction myelography (DSM) can be employed to find slow or intermittent leaks, often revealing the exact leak site as an active contrast extravasation or epidural fluid accumulation (Chung et al. 2000, Donovan et al. 2007, Sutton et al. 2022). In a recent case involving a thoracic spine manipulation, dynamic imaging (CT myelography) was required to identify the dural defect after static MRI sequences failed to do so (Bozer et al. 2024). Radionuclide cisternography is another modality that can demonstrate CSF leakage, though it is used less often in modern practice. In summary, the diagnosis of a post‐manipulation CSF leak rests on recognizing the characteristic clinical syndrome of orthostatic headache, corroborating it with MRI/LP findings of IH, and then utilizing targeted spinal imaging to locate the leak when intervention is being considered (Kusnezov et al. 2013, Chung et al. 2000, Jeret 2001, Beck et al. 2003, Suh et al. 2005, Strauss et al. 2005, Mathews et al. 2006, Morelli et al. 2006, Prasad et al. 2006, Donovan et al. 2007, Kurbanyan and Lessell 2008, Wagner et al. 2010, Ahn et al. 2013, Wilson et al. 2015, Hawson and Taylor 2015, Lin and Weng 2017, Fernando et al. 2022, Sutton et al. 2022, Bozer et al. 2024, Kim 2024, Chen et al. 2011).

In many cases of spontaneous spinal cord injury, the cause is a ventral dural tear in the thoracic spine, and MRI typically shows a ventral epidural fluid collection extending over multiple levels. This seems to hold true in post‐manipulation leaks as well: for example, the patient described by Kusnezov et al. (2013) had a C6‐ventral epidural fluid collection on MRI, and in the Bozer et al. (2024) case, a thoracic epidural fluid accumulation was eventually visualized at the leak site. These collections are typically isointense to CSF and may compress the thecal sac slightly, though without a true mass effect on the cord in most instances. Secondary spinal MRI signs include engorgement of epidural venous plexuses and, occasionally, indirect clues like a “false localizing” C1–C2 fluid collection, which can appear in some leaks irrespective of the actual tear location (Suh et al. 2005). If a meningeal diverticulum is present and ruptured, one might see an irregular outpouching or fluid at a nerve root sleeve. MRI myelography can enhance the contrast between CSF and adjacent tissues, facilitating the detection of such leaks. In subtle cases, CT myelography is often more sensitive, showing active leakage of contrast out of the intrathecal space or pooling of contrast in the epidural space. Some leaks, particularly CSF‐venous fistulas, do not produce a sizable fluid collection; instead, contrast may wash out rapidly into a venous structure. In challenging cases, DSM performed with the patient in the lateral decubitus or Trendelenburg position can dynamically identify intermittent CSF leaks or fistulas by visualizing rapid contrast passage and opacification (Bozer et al. 2024).

In post‐manipulation cases with CSF leak, there is usually no gross osseous injury visible. Thus, standard spine x‐rays or even routine MRI might appear normal except for the subtle fluid sign (Chung et al. 2000, Prasad et al. 2006, Wilson et al. 2015, Chen et al. 2011). Therefore, the imaging workup for a suspected CSF leak after manipulation should include a brain MRI to confirm IH signs, followed by dedicated spinal imaging (MRI myelography or CT myelography) to find the leak site.

### 4.3 Management Strategies

Management of CSF leaks and IH after SMT aligns with the standard treatment approach for spontaneous CSF leaks. There are conservative, minimally invasive, and surgical options, and an individualized stepwise strategy is generally adopted based on symptom severity and the leak's response to initial therapy.

Many cases of IH improve spontaneously with time and rest, as the dural rent can heal on its own. Initial treatment, therefore, involves conservative measures such as strict bed rest, as horizontal posture minimizes pressures, adequate hydration, caffeine intake, and analgesics for pain relief (Tuchin 2014). In the case series and reports on post‐manipulation leaks, this conservative approach has often been the first‐line (Tuchin 2014). For example, Kusnezov et al. (2013) reported managing their patient with 2 weeks of bed rest and observed a complete spontaneous recovery. A follow‐up MRI at 6 months showed the previously noted CSF collection had significantly decreased, correlating with symptom resolution. Thus, a watch‐and‐wait approach is reasonable in cases that are mild and tolerable. During this period, careful monitoring is needed for any neurological worsening or development of complications.

If conservative measures fail to produce improvement after a few days to a week or if the patient's orthostatic headaches are incapacitating, an EBP is the treatment of choice (Ernst 2007, Fernando et al. 2022, Chen et al. 2011, Tuchin 2014, Chiapparini et al. 2004, Palermo et al. 2025). In the context of SIH, EBPs are a well‐established, low‐risk intervention and often result in rapid symptom relief (Romozzi, Garignano, Funcis, et al. 2025). The same approach may be applied to post‐manipulation leaks. In multiple reported cases, a single EBP resulted in marked clinical improvement.

A minority of cases will not respond to even repeated blood patching. If a debilitating headache persists and imaging continues to show an active CSF leak, surgical intervention may be required for a definitive fix (laminectomy or laminotomy at the level of the leak, identifying the dural defect, and suturing or sealing it). Surgery is especially indicated if an underlying spinal pathology is contributing (Wilson et al. 2015, Bozer et al. 2024).

All patients with IH, regardless of cause, should be monitored for complications. One potential complication of a prolonged low‐pressure state is the development of subdural hematomas from torn bridging veins that may require urgent neurosurgical evacuation. Patients with SIH due to CSF leak exhibit a remarkably higher prevalence of subdural hematomas, ranging from 25.9% to 43.5% (Overstijns et al. 2025). The suspected pathomechanism is that the reduced intracranial CSF volume leads to brain sagging and thus tearing of bridging veins. They are typically bilateral but often asymmetric, and the maximal thickness is commonly reported in the 1–3 cm range with variable degrees of mass effect and occasional midline shift (Girão et al. 2018). Another important complication of SIH is cerebral venous thrombosis (CVT), with a reported incidence of approximately 2% in SIH (Schievink and Maya 2008). Proposed mechanism to explain this phenomenon involves engorgement of venous structures results in the slowing of venous blood flow, sagging of the brain that causes traction on cerebral veins and sinuses, possible reduction of CSF absorption into the cerebral venous sinuses as seen in SIH, leading to an increase in blood viscosity (Girão et al. 2018, Zhang et al. 2018). In the case we reported and in some post‐manipulative cases from the literature, patients presented focal deficits from subdural hematomas or brain sagging, necessitating intensive care and simultaneous treatment of the CSF leak (Prasad et al. 2006, Wilson et al. 2015, Lin and Weng 2017). The acute presentation of the condition with a subdural hematoma as a complication further underscores the critical importance of accurately establishing the potential etiological association between the precipitating maneuvers and the onset of IH. Managing the hematoma alone, without addressing the underlying hypotension, may result in recurrence, as observed in our case, or in more severe complications such as nerve sheath tear of neurological deficits.

This scoping review has several limitations. First, the available evidence is based almost entirely on isolated case reports, which are subject to publication and reporting biases and do not allow causal inference or estimation of incidence. Second, a temporal relationship between spinal manipulation and symptom onset may be influenced by recall bias, and important clinical details (including the exact maneuver performed, operator expertise, preexisting spinal abnormalities, connective tissue features, and prior headache history) were inconsistently reported across studies. Third, diagnostic work‐up and imaging protocols were heterogeneous, and leak localization was not uniformly pursued with advanced myelographic techniques, limiting comparisons across cases. Fourth, treatment strategies and follow‐up durations varied widely; therefore, apparent response rates—particularly for EBP—should be interpreted cautiously and not as estimates of effectiveness.

## 5 Conclusion

The incidence of spinal manipulation‐associated CSF leaks is not known. This condition should be suspected when patients present with a new‐onset headache, especially orthostatic, or with worsening of a primary headache disorder, after manual treatment. Diagnosis is primarily based on MRI, with advanced myelography occasionally employed to localize the CSF leak. Management is largely conservative and minimally invasive, with EBP being highly effective in most cases, and surgery reserved for refractory cases or those with identifiable lesions. With timely intervention, the prognosis is generally favorable.

Moving forward, increased awareness and documentation will help delineate risk factors for this complication. However, the presence of preexisting degenerative spinal pathology, especially cervical spondyloarthrosis, along with the history of spontaneous CSF leaks or connective tissue disorders, should be carefully assessed by chiropractors and other manual therapists. Professionals performing spinal manipulation may consider appropriate patient selection and follow‐up.

## Author Contributions

Conception and design: Matteo Palermo, Marina Romozzi, and Francesco Signorelli. Data collection: Giuseppe Garignano, Marina Romozzi and Francesco Signorelli. Data analysis: Matteo Palermo, Fabio Zeoli, and Marina Romozzi. Drafting: Matteo Palermo, Marina Romozzi, Francesco Signorelli, and Federico Tosto. Draft revision: Matteo Palermo, Marina Romozzi, Francesco Signorelli, Federico Tosto and Catello Vollono.

## Funding

The authors have nothing to report.

## Ethics Statement

The authors have nothing to report.

## Consent

The patient reported in the case report has provided written informed consent.

## Conflicts of Interest

The authors declare no conflicts of interest.

## Supporting information



Supplementary Figure. Sagittal T2‐weighted fat‐suppressed image demonstrating a posteriorly located extradural collection extending from the C4 level to L1, characterized by mixed signal intensity consistent with fluid admixed with hemorrhagic components.

## Data Availability

Rough data are available upon request.
