# Supplementary figures and images for: Intracranial Hypotension Following Spinal Manipulation: A Case Report and Scoping Review of the Literature
Source: Brain Behav. 2026 Apr 27;16(5):e71409. doi: 10.1002/brb3.71409 (PMC13118394; doi:10.1002/brb3.71409)

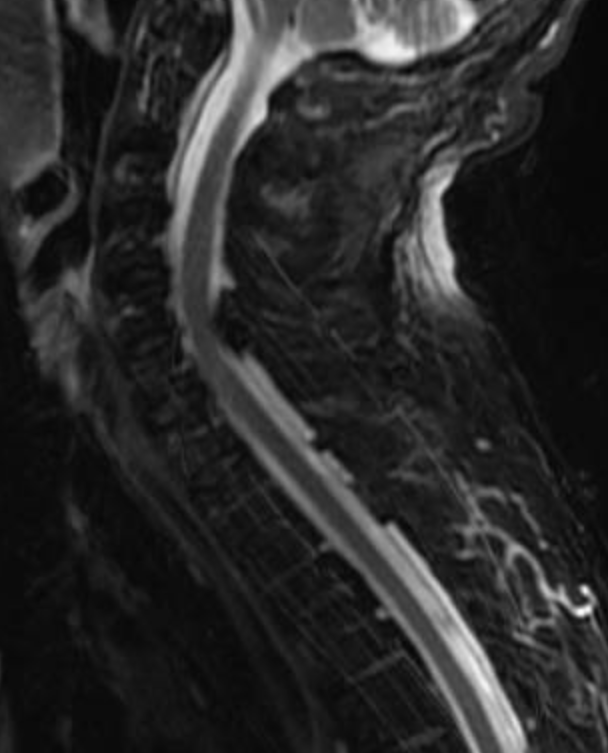

Supplement: Supplementary file 1 — Supplementary Figure. Sagittal T2‐weighted fat‐suppressed image demonstrating a posteriorly located extradural collection extending from the C4 level to L1, characterized by mixed signal intensity consistent with fluid admixed with hemorrhagic components. [file BRB3-16-e71409-s001.png]
